# Exploring the quality of life of older people in long-term care facilities in the Sub-Saharan region: a scoping review

**DOI:** 10.1186/s12877-025-06437-z

**Published:** 2025-10-28

**Authors:** Naomi Hlongwane, Lieketseng Ned

**Affiliations:** 1 Division of Disability and Rehabilitation Studies, Department of Global Health, Faculty of Medicine & Health Sciences, Stellenbosch University, Cape Town, South Africa; 2 Division of Disability and Rehabilitation Studies, Department of Global Health, Faculty of Medicine & Health Sciences, Stellenbosch University, Cape Town, South Africa

**Keywords:** Long term care, Quality of life, Sub-Saharan africa, Older people, Policy

## Abstract

**Background:**

Over the past decade, there has been a shift in the family-centered model of care for older people in Sub-Saharan African countries, driven primarily by urbanization and industrialization. As the aging population grows, there is an increasing need for long-term care. However, limited evidence exists on the quality of life of older people in long-term care facilities across the region. This scoping review aims to describe the existing evidence on the quality of life of older people living in long-term care facilities in Sub-Saharan Africa.

**Methods:**

A systematic search was conducted from 26 March 2024 to 15 July 2024 on four databases: EBSCOHOST, PubMed, Scopus, Web of Science, and grey literature was included as an additional source of information. This study follows the methodological framework as presented by Arksey and O’Malley and incorporating Levac and colleagues’ recommendations. A total of 360 peer-reviewed articles were identified and screened using CADIMA software. Studies were included if they focused on the quality of life of older people in long-term care facilities in sub-Saharan Africa, were published in English, and involved participants aged 60 and above. Twenty-one articles met the inclusion criteria and were analyzed using data charting process through thematic analysis which involved identifying, synthesizing, and interpreting key themes across the studies to address the review objective.Bronfenbrenner’s ecological systems theory was applied to guide the interpretation and organization of findings, focusing on five interconnected themes: microsystem, mesosystem, exosystem, macrosystem, and chronosystem.

**Results:**

Findings showed underrepresentation of articles from different countries with the 21 studies, spanning five sub-Saharan African countries,and the majority being from South Africa. These studies utilized qualitative approaches such as case studies and phenomenological research. Key tools included interviews and quality of life scales. Most studies were conducted in privately funded long-term care facilities, emphasizing the influence of gender, ethnicity, socio-economic status, and education on quality of life. Findings indicated that multiple factors at different ecological levels (microsystem to chronosystem) affect quality of life in long-term care.

**Conclusions:**

Evidence shows a lack of structured long-term care policy in sub-Saharan Africa. Given the increasing demand for long-term care, there is an urgent need for policies that address factors affecting quality of life and ensure better care for older people in these facilities. A protocol for the scoping review was preregistered on the Open Science Framework Registry on 9th September 2023 (https://osf.io/xm8r5/).

**Supplementary Information:**

The online version contains supplementary material available at 10.1186/s12877-025-06437-z.

## Introduction

The rapid increase in the global geriatric population has led to a significant demographic shift, with the proportion of the world’s population aged 60 and above experiencing a rapid growth rate. This trend is evident worldwide, impacting both low- and middle-income countries (LMICs) and high-income countries (HICs) alike [1]. The number of people aged 65 and over is projected to double by 2050, reaching approximately 1.5 billion, an increase of roughly 16%. Africa has a relatively small geriatric population compared to other continents, with only around 5% of the population considered aged 60 and above, however an increase in this population is anticipated. In sub-Saharan Africa, the older population is estimated to reach 67 million by 2025 and 163 million by 2050 [2]. Sub-Saharan Africa faces numerous challenges in providing adequate care and support for its older population, especially in long-term care (LTC) settings. Understanding the issues encountered by older people, and their impact on quality of life (QoL), is essential for developing effective care models [3, 4]. In traditional African societies and other low-income regions, care for older people has been largely provided by extended family networks within the respective households [5]. Older people are known to reside with family especially in rural areas where there is minimal access to LTC facilities to care for them as their health declines. However, studies conducted in the last decade show that there is a change and transition in the family centred model of care for older people within sub-Saharan African countries [6, 7]. Factors driving this change include increased rural-to-urban migration and employment rates, particularly among young women, the impact of the HIV/AIDS epidemic, and easing of beliefs and norms in society [8]. The World Health Organization (WHO) defines LTC as activities undertaken by others to ensure that individuals with significant ongoing loss of intrinsic capacity can maintain functional ability consistent with their basic rights, fundamental freedoms, and human dignity [9]. This definition emphasizes the importance of QoL, including nutrition, rehabilitation, and physical activity. The WHO’s global strategy on aging and health reaffirms the promotion of healthy aging and the maintenance of functional ability [10]. Furthermore,WHO [11] defines QoL as a multidimensional and subjective concept, describing it as the individual’s perception of their position in life in the context of the culture and value systems in which they live, and in relation to their goals, expectations, standards, and concerns. Developing measurement instruments that reflect the multidimensionality of QoL is crucial, and several scales have been created for this purpose. QoL is commonly assessed through multidimensional tools such as Flanagan’s domain model and the WHOQOL-BREF, which emphasize physical, psychological, social, and environmental well-being [12–15]. While instruments like the WHOQOL-OLD have been validated for older populations in South Africa and show strong reliability. Broader reviews highlight persistent gaps in the cultural sensitivity, psychometric quality, and accessibility of QoL measurements particularly for older people living in resource-constrained or institutional settings [16]– [17].

There is a close relationship between older people and their environment and its impact, not only on their health status, but on QoL [18]. Studies conducted on QoL among older people show that, when older people can live and receive support in their preferred care location, they report a better QoL [19, 20]. However, those living in LTC facilities have been associated with poorer well-being and QoL [21].

The QoL of older people within the African context is influenced by various factors. Studies highlight that dimensions such as household services, economic status, safety, and mental and physical health play significant roles in determining the well-being of older people [22]. Additionally, social, economic, health, and environmental factors, along with the prevalence of non-communicable diseases and geriatric syndromes, impact the QoL of older people in Africa [23, 24]. Furthermore, difficulties in performing activities of daily living (ADL) have been shown to have a detrimental effect on health and QoL among older individuals in South Africa and Uganda [25].

Research also shows that the relocation process to LTC facilities impacts both the QoL and subjective well-being of older people [26–28]. Shin and Park highlight the significance of adjustment to facility living, self-esteem, subjective health, interpersonal relationships, and the level of care in influencing QoL [29]. The standard of care within LTC is associated with the level of training and support for care workers. A study conducted by Mapira and colleagues [30] shows that the experiences of care workers in LTC facilities are influenced by inadequate training, policy support, role ambiguity, poor employment conditions, and lack of career growth opportunities, all of which impact the quality of care provided to older people.

Despite existing LTC policies and social welfare programs, barriers to achieving good QoL persist [31, 32]. Efforts to improve QoL for older people in LTC in sub-Saharan Africa must address the above-mentioned factors, including healthcare access, social support, poverty alleviation, and cultural sensitivity. Sustainable development and investments in healthcare infrastructure and community-based support systems are vital for enhancing the well-being of the elderly population in the region.

To contribute to the development of effective QoL models for LTC, it is essential to obtain reliable measurement instruments and gain an understanding of the QoL of older people within sub-Saharan Africa, where LTC is not well-funded, prioritized and standardized in terms of care models and policy.

This scoping review thus seeked to use Institutional Ethnography (IE) and Bronfenbrenner’s ecological systems theory as lenses to bring to light the importance of ‘macrosystems’ in the formation of low-level interactions with older people’ QoL. IE seeks to understand how the social organization of institutions impacts the day-to-day activities of marginalized groups and the meaning they attach to their experiences [33]. Bronfenbrenner’s ecological systems theory provides a robust theoretical framework for understanding the factors associated with the QoL of older people by exploring the interactions between individual, relational, and societal factors. This model emphasizes that QoL is shaped by multiple layers of environmental contexts, ranging from immediate family and caregivers to societal structures and cultural norms. The scoping review will employ this theory to investigate how these layered systems influence the QoL of older people in LTC.

The first level, the microsystem, which involves direct interactions within the LTC facility – such as relationships with caregivers, other residents, and facility conditions – has been shown to have a significant impact on QoL. Research by Shin and Park demonstrates that facility-based environmental factors explain up to 83% of QoL variations, underscoring the critical role that immediate surroundings play [34].

The second level is the mesosystem, incorporating the connections and interactions between different microsystem elements (e.g., relationships between older residents and their families, and between residents and caregivers). These interactions determine the support older people receive, both emotionally and socially, and contribute to their sense of belonging. The third level is the exosystem, which includes broader social structures, such as LTC policies and regulatory frameworks, and indirectly impacts older people by shaping the quality of care provided. For instance, Genienė et al. highlight the role of community-based services in deinstitutionalizing care, demonstrating how policy decisions can enhance QoL by promoting community engagement and support [35]. At the macrosystem level, cultural attitudes towards aging, such as ageism, operate across all layers of Bronfenbrenner’s framework. Gendron et al. emphasize that addressing ageism requires interventions at the macrosystem level [36]. Bronfenbrenner’s ecological systems theory provides a holistic view of how different levels of the environment interact to shape the experiences and QoL of older people. The rationale for using this model in the scoping review is the model’s ability to contextualize individuals within interlocking social systems that influence each other across levels. As Snyder suggests, this theory allows for an understanding of the quality and nature of interactions between individuals and their support systems [37].

Ethnographic research has studied both individual (microsystems) and relational (mesosystem) interactions in understanding QoL, but has neglected the macrosystem [38–41]. Within the context of this review, using IE will assist in demonstrating how the general society influences the day-to-day QoL of older people through its policies, unwritten rules and practices. The Bronfenbrenner ecological systems theory lens will assist in the interpretation of data to aid an understanding of the interactions between the societal, organizational and individual dimensions, and will highlight the constructs of QoL of older people in LTC facilities in relation to organizations and society. The Bronfenbrenner ecological systems theory will guide the kind of data to be collected from all the levels described in the model and, in addition, inform the analysis process [42, 43]. Both these frameworks bring value to the review because they acknowledge how organizations shape the lives of older people in society.

The research question for this review was to describe the existing evidence on the quality of life of older people living in long-term care facilities in Sub-Saharan Africa.

## Materials and methods

### Design

This review followed the methodological framework as presented by Arksey and O’Malley [44] and incorporating Levac and colleagues’ [45] recommendations. All five steps of the scoping review framework were followed: (a) confirming the primary research question; (b) identifying the related studies through a systematic search; (c) study selection; (d) extracting key information and creating a data-charting form; and (e) collating, summarizing, and presenting the results. This scoping review seeked to answer broad questions systematically, with a rigorous, transparent and reliable synthesis of knowledge guided by the Preferred Reporting Items for Systematic Reviews and Meta-analyses Extension for Scoping Reviews (PRISMA-ScR) [46]. The choice of this method is anchored in the need for extensive mapping of the literature on this emerging topic., 

### Step 1: confirming the primary research question

The following research questions were developed according to the PCC (Population, Concept, Context) as shown in Table 1 below.

**Table 1:** PCC framework

*P*: Population	Older people: This includes individuals aged ≥ 60 years
C: Concept	Quality of life: QoL refers to an individual’s overall well-being and satisfaction with various aspects of their life, including physical, psychological, social, and environmental dimensions. This study will adopt the WHO definition of QoL which states that QoL is, “how an individual views their position in life in the setting of cultural value systems in which they live in relation to their goals, expectations, standards and concerns. It is an expansive term that includes physical health, psychological state, independence level, social relationships, personal beliefs and how an individual relates to the environment surrounding them” (p.1405) [7]. Long-term care facility: The WHO argues that all countries should have a long-term care system, defining these broadly as: “Activities undertaken by others to ensure that people with or at risk of a significant ongoing loss of intrinsic capacity can maintain a level of functional ability consistent with their basic rights, fundamental freedoms and human dignity” (p.2) [47].
C: Context	sub-Saharan Africa Sub-Saharan Africa is geographically the area of the African continent that lies south of the Sahara Desert. It includes 48 countries of which 23 are low-income.

The broad research question was: “To date, what evidence exists on the QoL in LTC facilities among geriatric populations in sub-Saharan Africa?

The sub questions for the review were:

1. What is the current state of knowledge regarding QoL in LTC facilities among older people in sub-Saharan Africa?
2. What factors influence the QoL of older people in LTC facilities ?
3. What measurement tools have been used to assess the QoL in this population in LTC facilities ?

The CADIMA web tool was used to conduct the scoping review. This tool ensures an automated allocation of records during the process and assists the authors in the question formulation, protocol development, duplicate checking, automated allocation of records during the screening process, study selection and documentation. A protocol for the scoping review was preregistered on the Open Science Framework Registry on 9th September 2023 (https://osf.io/xm8r5/*).*

### Step 2: Identifying relevant studies and developing a search strategy

The database search query was composed of the above-mentioned three search concepts of PCC. The final search terms and string were derived from a preliminary search and analyzed by comparing the words found in titles, abstracts and keywords. The PCC acronym was used to define key concepts for this study. Additionally, to enhance the accuracy and comprehensiveness of the search results, all authors were involved in a consensus process, and an additional librarian expert was consulted to validate the identified search terms and suggest any additional relevant keywords.

A comprehensive search was performed from 26 March 2024 until 15 July 2024 across four electronic databases: EBSCOHOST, PubMed, Scopus, and Web of Science, to identify published literature surrounding the research question from the inception date of the database to 15 July 2024. Grey literature was included as an additional source of data. The appendices (Table 2) displays the search string example used in each database and the number of studies found.

**Table 2:** Search string results

Search string	Database source	#results (#origin)
(aged OR elderly OR “Old people” OR “Old person” OR Elders OR “Elderly people” OR “Elderly person” OR Seniors) AND (“Elderly population” OR “home old age” OR “Geriatric Long-Term Care Facilities” OR “Homes for the Aged” OR “Nursing Home” OR “Continuing Care Retirement Centers” OR “Housing for the elderly” OR “home for the elderly” OR “Institutionalized older adults” OR “Institutionalized elderly” OR “old age homes” OR “care home” OR “retirement home” OR “residence of age care” OR “geriatric home” OR “geriatric care” OR “residential care home”) AND (“Health-Related Quality of Life” OR HRQoL OR “Quality of life” OR wellbeing OR “well-being” OR “life quality”) AND (Angola OR Benin OR Botswana OR Bobo-Dioulasso OR “Burkina Faso” OR Burundi OR Cameroon OR “Cape Verde” OR “Central African Republic” OR Chad OR Comoros OR Congo OR Brazzaville OR “Cote d’Ivoire” OR “Ivory Coast” OR Djibouti OR “Equatorial Guinea” OR Eritrea OR Ethiopia OR Gabon OR Gambia OR Ghana OR Guinea OR Bissau OR Kenya OR Lesotho OR Liberia OR Madagascar OR Malawi OR Mali OR Mauritania OR Mauritius OR Mozambique OR Namibia OR Niger OR Nigeria OR Rwanda OR “Sao Tome e Principe” OR Senegal OR Seychelles OR “Sierra Leone” OR Somalia OR “South Africa” OR “south sudan” OR Sudan OR Swaziland OR Tanzania OR Togo OR Uganda OR “western sahara” OR Zaire OR Zambia OR Zimbabwe OR “Africa South of the Sahara” OR “Sub-Saharan Africa” OR “Southern Africa” OR “subsaharan Africa” OR “sub sahara” OR subsahara OR Africa)	Scopus	69 (115)
(aged OR elderly OR “Old people” OR “Old person” OR Elders OR “Elderly people” OR “Elderly person” OR Seniors) AND (“Elderly population” OR “home old age” OR “Geriatric Long-Term Care Facilities” OR “Homes for the Aged” OR “Nursing Home” OR “Continuing Care Retirement Centers” OR “Housing for the elderly” OR “home for the elderly” OR “Institutionalized older people” OR “Institutionalized elderly” OR “old age homes” OR “care home” OR “retirement home” OR “residence of age care” OR “geriatric home” OR “geriatric care” OR “residential care home”) AND (“Health-Related Quality of Life” OR HRQoL OR “Quality of life” OR wellbeing OR “well-being” OR “life quality”) AND (Angola OR Benin OR Botswana OR Bobo-Dioulasso OR “Burkina Faso” OR Burundi OR Cameroon OR “Cape Verde” OR “Central African Republic” OR Chad OR Comoros OR Congo OR Brazzaville OR “Cote d’Ivoire” OR “Ivory Coast” OR Djibouti OR “Equatorial Guinea” OR Eritrea OR Ethiopia OR Gabon OR Gambia OR Ghana OR Guinea OR Bissau OR Kenya OR Lesotho OR Liberia OR Madagascar OR Malawi OR Mali OR Mauritania OR Mauritius OR Mozambique OR Namibia OR Niger OR Nigeria OR Rwanda OR “Sao Tome e Principe” OR Senegal OR Seychelles OR “Sierra Leone” OR Somalia OR “South Africa” OR “south sudan” OR Sudan OR Swaziland OR Tanzania OR Togo OR Uganda OR “western sahara” OR Zaire OR Zambia OR Zimbabwe OR “Africa South of the Sahara” OR “Sub-Saharan Africa” OR “Southern Africa” OR “subsaharan Africa” OR “sub sahara” OR subsahara OR Africa)	Web of Science	14 (46)
(aged OR elderly OR “Old people” OR “Old person” OR Elders OR “Elderly people” OR “Elderly person” OR Seniors) AND (“Elderly population” OR “home old age” OR “Geriatric Long-Term Care Facilities” OR “Homes for the Aged” OR “Nursing Home” OR “Continuing Care Retirement Centers” OR “Housing for the elderly” OR “home for the elderly” OR “Institutionalized older people” OR “Institutionalized elderly” OR “old age homes” OR “care home” OR “retirement home” OR “residence of age care” OR “geriatric home” OR “geriatric care” OR “residential care home”) AND (“Health-Related Quality of Life” OR HRQoL OR “Quality of life” OR wellbeing OR “well-being” OR “life quality”) AND (Angola OR Benin OR Botswana OR Bobo-Dioulasso OR “Burkina Faso” OR Burundi OR Cameroon OR “Cape Verde” OR “Central African Republic” OR Chad OR Comoros OR Congo OR Brazzaville OR “Cote d’Ivoire” OR “Ivory Coast” OR Djibouti OR “Equatorial Guinea” OR Eritrea OR Ethiopia OR Gabon OR Gambia OR Ghana OR Guinea OR Bissau OR Kenya OR Lesotho OR Liberia OR Madagascar OR Malawi OR Mali OR Mauritania OR Mauritius OR Mozambique OR Namibia OR Niger OR Nigeria OR Rwanda OR “Sao Tome e Principe” OR Senegal OR Seychelles OR “Sierra Leone” OR Somalia OR “South Africa” OR “south sudan” OR Sudan OR Swaziland OR Tanzania OR Togo OR Uganda OR “western sahara” OR Zaire OR Zambia OR Zimbabwe OR “Africa South of the Sahara” OR “Sub-Saharan Africa” OR “Southern Africa” OR “subsaharan Africa” OR “sub sahara” OR subsahara OR Africa)	EBSCOHOST	103 (143)
aged[MeSH Terms] OR aged OR elderly OR “Old people” OR “Old person” OR Elders OR “Elderly people” OR “Elderly person” OR Seniors) AND (nursing homes[MeSH Terms] OR Housing for the elderly[MeSH Terms] OR Homes for the Aged[MeSH Terms] OR “Elderly population” OR “home old age” OR “Geriatric Long-Term Care Facilities” OR “Homes for the Aged” OR “Nursing Home” OR “Continuing Care Retirement Centers” OR “Housing for the elderly” OR “home for the elderly” OR “Institutionalized older people” OR “Institutionalized elderly” OR “old age homes” OR “care home” OR “retirement home” OR “residence of age care” OR “geriatric home” OR “geriatric care” OR “residential care home”) AND (Quality of life[MeSH Terms] OR “Health-Related Quality of Life” OR HRQoL OR “Quality of life” OR wellbeing OR “well-being” OR “life quality”) AND (Africa South of the Sahara[MeSH Terms] OR Angola OR Benin OR Botswana OR Bobo-Dioulasso OR “Burkina Faso” OR Burundi OR Cameroon OR “Cape Verde” OR “Central African Republic” OR Chad OR Comoros OR Congo OR Brazzaville OR “Cote d’Ivoire” OR “Ivory Coast” OR Djibouti OR “Equatorial Guinea” OR Eritrea OR Ethiopia OR Gabon OR Gambia OR Ghana OR Guinea OR Bissau OR Kenya OR Lesotho OR Liberia OR Madagascar OR Malawi OR Mali OR Mauritania OR Mauritius OR Mozambique OR Namibia OR Niger OR Nigeria OR Rwanda OR “Sao Tome e Principe” OR Senegal OR Seychelles OR “Sierra Leone” OR Somalia OR “South Africa” OR “south sudan” OR Sudan OR Swaziland OR Tanzania OR Togo OR Uganda OR “western sahara” OR Zaire OR Zambia OR Zimbabwe OR “Africa South of the Sahara” OR “Sub-Saharan Africa” OR “Southern Africa” OR “subsaharan Africa” OR “sub sahara” OR subsahara OR Africa	Pubmed	26 (44)
elderly “old age home” and “well-being” Africa	Grey literature	11 (12)

### Step3: study selection

All titles and abstracts obtained in the search were independently screened by two reviewers (NH and ZG). Discrepancies were resolved by a third reviewer (LN). Twenty one studies were included for full-text review based on the following criteria (Table 3):

**Table 3:** Eligibility criteria

Inclusion criteria	Exclusion criteria
¬ Electronic database, reference list of included studies existing in Google website.	
¬ Individuals ≥ 60 years old.	¬ Individuals younger than 60 years.
¬ Focus on QoL or related constructs (e.g., well-being, life satisfaction).	
¬ QoL in LTC facilities, such as nursing homes, residential care facilities.	¬ Hospitals, home care settings, mental health facilities, and subacute and acute facilities.
¬ Published peer-reviewed studies conducted in sub-Saharan Africa.	¬ Studies conducted outside sub-Saharan Africa.
¬ Publications in English.	¬ Publications in other languages such as French, Arabic, Chinese, and others.
¬ Primary study designs, such as quantitative, qualitative, and mix-method studies with human participants as well as guidelines. Other review studies, such as systematic, literature and scoping reviews.	

### Step 4: data extraction

Two reviewers (NH and ZG) gathered the study characteristics and main outcome of each study. Only studies that met all inclusion criteria were included in the final text review. Data charting was then performed on the final set of included studies using a standardized charting form. This process involved systematically extracting key information such as study characteristics, methodological approach, population details, quality of life domains assessed, and main findings. The charting process was iterative and guided by the review objectives, with adjustments made as needed to capture relevant themes and patterns.

Any discrepancies were resolved through consensus via a joint review between the reviewers.

### Step 5: synthesis of results

Thematic synthesis was employed to analyze the results, with themes developed inductively from the data. This approach allowed for the identification of patterns and trends across the included studies. The data were synthesized describing the aim, methodology, sample, and the main outcomes of each study. After the selection of studies, a thematic analysis was conducted using the thematic synthesis protocol. This involved organizing the findings in themes and sub-themes and abstracting the relevant information regarding the description, dimensions, and factors that contribute to the QoL of older people living in LTC within the sub-Saharan region.

Key themes were discussed in multiple sessions to ensure consensus was reached. As the key themes included a diverse range of variables at different levels, the reviewers came to a consensus to code the data, integrate the codes into categories, create overarching themes, and apply these to a socio-ecological framework.

## Results

### Selection process

**Fig. 1:**
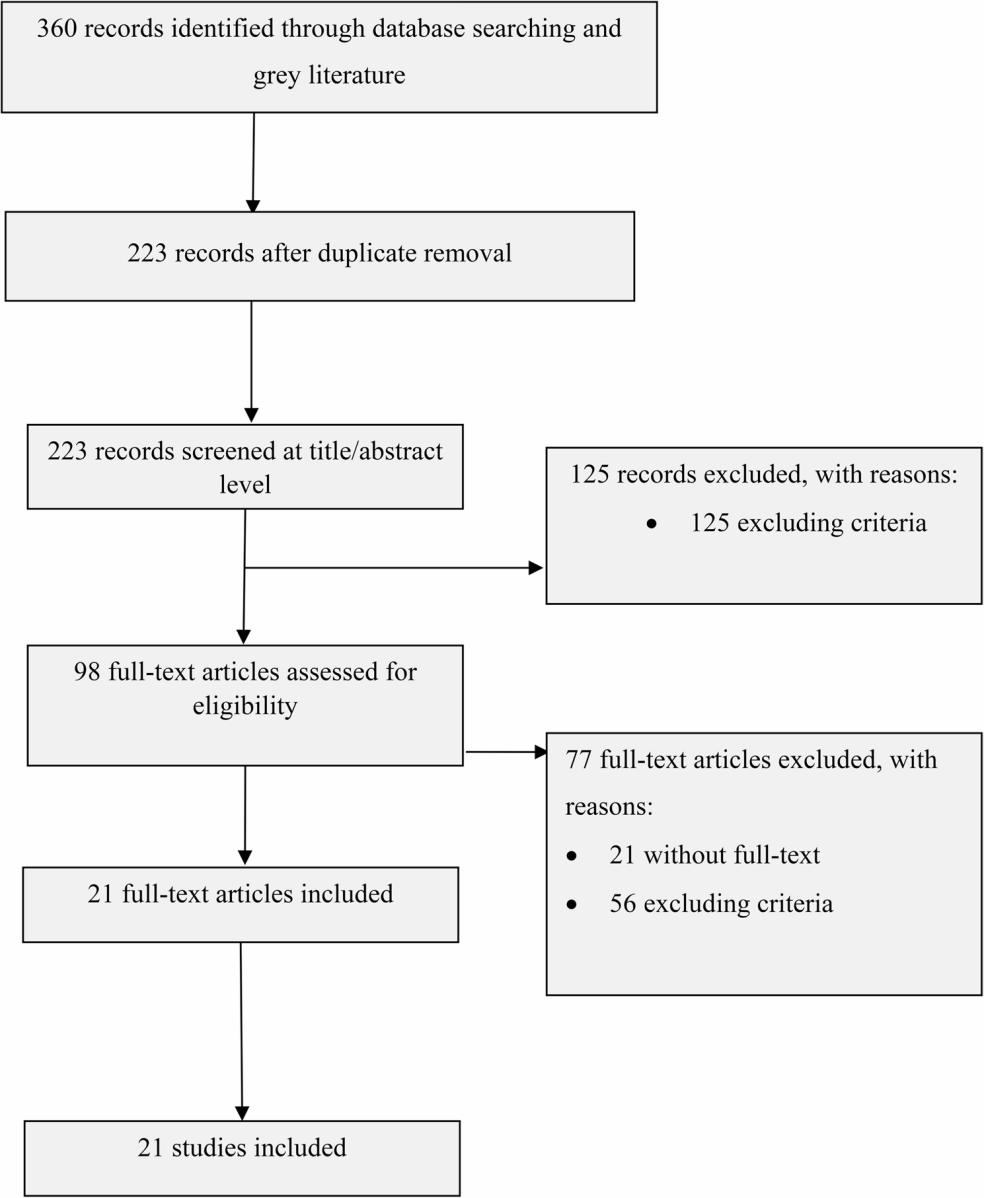
Reporting items for systematic reviews and meta-analysis (PRISMA-Scr) flow chart

The study selection process is illustrated in the PRISMA-ScR flow diagram (see Fig. 1). A total of 360 records were identified through comprehensive database searches. After the removal of duplicates, 223 records remained and were screened by two independent reviewers based on titles and abstracts using CADIMA software. Discrepancies between reviewers were resolved through discussion and consensus. Following this initial screening, 125 records were excluded for not meeting the inclusion criteria. The remaining 98 articles underwent full-text review. Of these, 77 articles were excluded—21 due to the unavailability of full texts and 56 for not meeting the eligibility criteria. Consequently, 21 studies were included in the final synthesis. Efforts were made to retrieve full texts of inaccessible articles by contacting corresponding authors, searching institutional repositories, and utilizing interlibrary loan services. Despite these efforts, some articles could not be accessed due to unresponsive authors or lack of publicly available copies. This limitation may have led to the exclusion of potentially relevant studies.

### Characteristics of the included studies

The 21 included studies cover the period from the inception date of the database to July 2024, and span five different countries (Ethiopia, South Africa, Nigeria, Angola, and Kenya) within the sub-Saharan region. A majority of the studies (*n* = 14) were based in South Africa with the other countries being Nigeria, Angola, Ethiopia and Kenya. The studies used a variety of study designs, inclusive of qualitative case study methodologies, phenomenological approaches, and interpretative phenomenological analysis (IPA) to explore various aspects of QoL among older people in care facilities across different African countries.

Key data collection tools included in-depth interviews, storytelling, observation checklists, and semi-structured interviews. Several validated questionnaires were also employed, such as the World Health Organization Quality of Life (WHOQoL) questionnaire, WHOQOL-OLD (an Older people WHOQOL module), the Satisfaction with Life Scale, and the Perceived Health Scale. Additionally, the Six-Dimensional EuroQol questionnaire (EQ-6D) and the ICEpop capability measure for older people (ICECAP-O) were used for assessing QoL. The studies also made use of the WHO (Five) Wellbeing Index (WHO-5), Kessler-6 (K6), and OSLO-3 Social Support Scale, along with the Ryff’s Psychological Well-being Scale (RPWBS), the University of California Loneliness Scale (UCLA-LS), and the RAND 36-Item Health Survey. Structured questionnaires captured personal information, physical activity, and attitudes, while other tools, such as the Life Satisfaction Index A, Likert Scale questionnaires, and productivity and generativity indicators, were also applied.

**Fig. 2:**
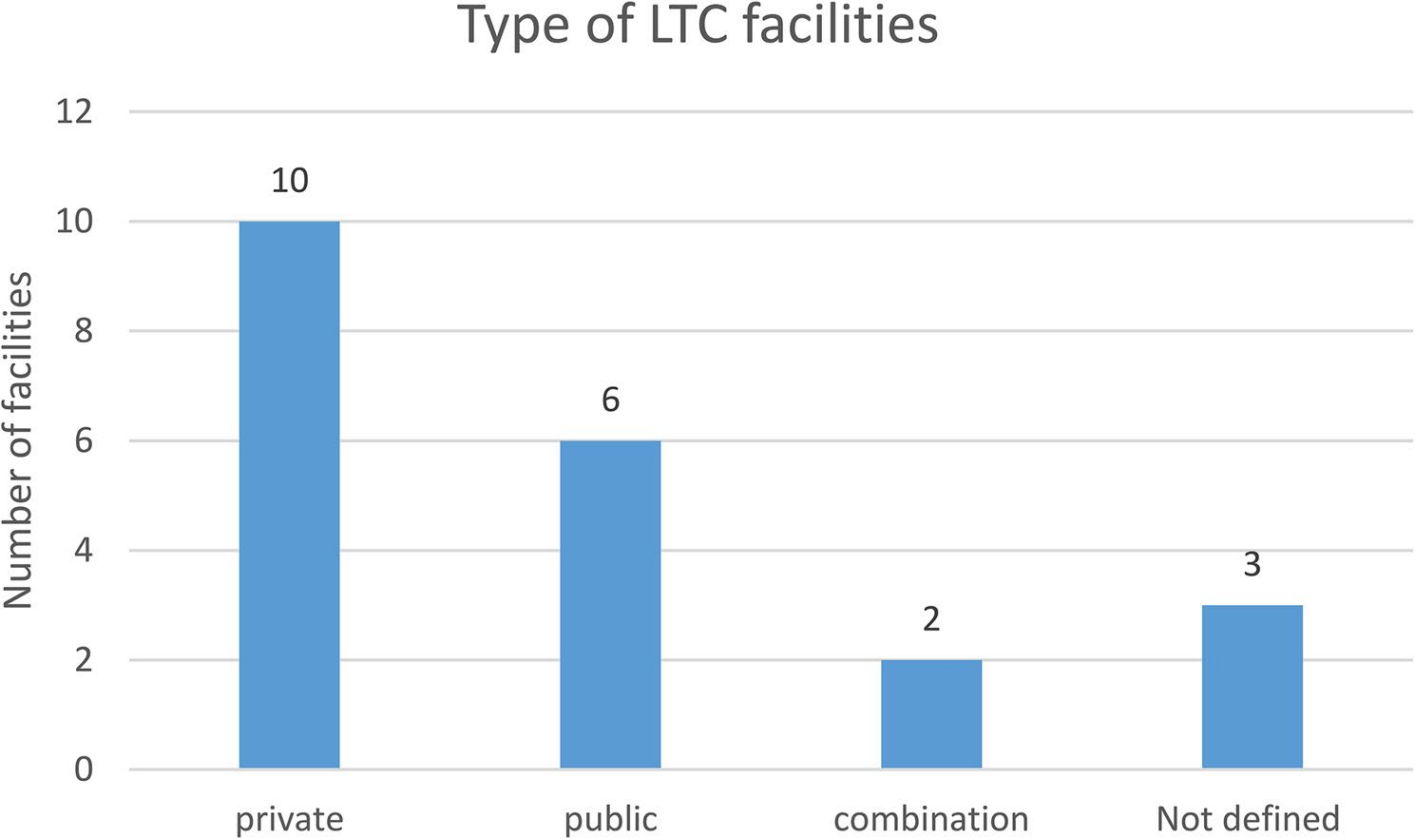
Type of LTC facilities

Out of the total (*N* = 21), majority of the studies (*n* = 10) were conducted in privately funded LTC facilities (see Fig. 2 below). The majority of participants in these studies were older females, above 65 years of age. Ethnic diversity was a critical factor, with some studies conducted in South Africa focusing on predominantly White participants [48, 49], while others include a significant proportion of Black South Africans or other ethnic groups [50, 51]. In addition to gender and ethnicity, socio-economic status and educational background influenced the findings. Some studies, such as Odetola et al. [52], focus on more affluent populations in well-established geriatric homes, while others explore populations in lower socio-economic conditions or publicly funded institutions, as in Chipps and Jarvis [53] and Teka and Adamek [54]. The variation in these factors related to issues of access to care, social capital, and overall well-being.

There were three main topic areas in the included studies. First, there were studies that describe or develop specific QoL models or scales for older people living in LTC. Secondly, there were studies that qualitatively explore perceptions of QoL both from stakeholders and older people. Lastly, there were studies that examine the relationships between QoL factors, some of which may serve as predictors of QoL. Four key interconnected themes related to the QoL of older people in LTC facilities were synthesized and categorized using Bronfenbrenner’s ecological systems theory, including (1) microsystem, (2) mesosystem, (3) exosystem, and (4) macrosystem (Table 4).

**Table 4:** Studies selected for scoping review

Author, year and title	Location of study	Type of facility	Aims	Study design	Sample size	Assessment tool	Main findings
Lizanle Van Biljon, Petrus Nel, & Vfera Roos 2015. *A partial validation of the WHOQOL-OLD in a sample of older people in South Africa*	South Africa	Private long term care facility	To partially validate the WHOQOL-OLD instrument by assessing its factor structure and internal consistency reliability in a South African context and to evaluate the psychometric properties of three short versions of the instrument.	Non-experimental, descriptive, cross-sectional survey design.	176 Afrikaans-speaking older people aged 61–95 years.	WHOQOL-OLD module (24-item, 6-facet instrument) and three short versions of the module.	The WHOQOL-OLD and its short versions demonstrated reliable psychometric properties in this South African sample. The original factor structure had good fit, while one short version (Version 3) showed the best fit for practical use. These findings support the use of the WHOQOL-OLD module for assessing quality of life in older people in long-term care settings.
Lizanlé Van Biljon & Vera Roos 2015. *The nature of quality of life in* *residential care facilities: the case of White older South Africans*	South Africa	Residential care facilities	To explore the perceptions of quality of life (QoL) among older people residing in residential care facilities in South Africa and provide insights for care providers.	Qualitative exploratory study using interpretative phenomenological analysis	41 older White South Africans aged 62–95 years (75% female).	Data were collected through semi-structured interviews, focus groups, and journal entries.	QoL was shaped by a spiritually-informed worldview, quality relationships, self-regulation, and the transitional nature of life experiences. Spirituality provided purpose, coping mechanisms, and mindfulness toward others. Relationships with family, close friends, and reciprocal interactions were central to QoL. QoL transitioned from a focus on “doing” and material achievements in younger years to “being” content and valuing meaningful experiences in later life. The findings highlight the dynamic and context-specific nature of QoL for older people in residential care settings.
Joseph, D.; van Niekerk, C. 2021. *Participative Musical Performance: Quality of Life at a Seniors’ Village in South Africa*	South Africa	Elite Helder-berg retirement village	Explore the benefits of community music and understand its impact on well-being in a retirement home.	Qualitative case study, phenomenological approach.	36 respondents (11 male, 25 female); age range: 60–90+	Questionnaire	Themes: Sense of Community, Desire to Learn, Spirituality. Music activities contribute significantly to life quality.
Jarvis, M., Ramlall, S.,Chipps, J. 2021. *A profile of social isolation and the influence of demographics in older* *persons living in residential care*,* Durban*,* South Africa*	South Africa	Non-private residential care facilities	To measure social isolation levels in older persons living in a lower socio-economic residential care setting in South Africa.	A cross-sectional survey was conducted using a	Oversampling resulted in a total of 384 residents. Residents aged 60 years and older, classified by the NPO as fit/independent, and without major sensory deficits, were eligible to participate.	Researcher administered questionnaire to measure the constructs of social isolation	Strong internal social networks within the facilities reduced loneliness through mutual support, trust, and reciprocity, despite low engagement in external community activities. Care models promoting interconnectedness (e.g., Ubuntu philosophy) can help mitigate social isolation. Policymakers and intake counselors should identify and support vulnerable residents, such as those struggling with integration or health issues. Psychiatric nurses must design programs addressing demographic shifts and promoting social engagement within facilities.
Odetola, T.D., Akintayo-Usman, N.O., Okorie, B.N., Afolabi, T.A., Oluwatosin, A.O. 2020. *Life Satisfaction Assessment of Elderly Living in Geriatric Homes: Case Study of a Geriatric Home in Ibadan*,* Nigeria.*	Nigeria	Government-registered geriatric home	To assess the life satisfaction of elderly residents in a geriatric home, identify factors affecting their satisfaction, determine reasons for their admission, and explore their perceptions of the home.	Descriptive, exploratory qualitative study	13 elderly participants aged 65–95 years.	In-depth interviews guided by a semi-structured interview tool, incorporating the Satisfaction with Life Scale (SWLS).	The participants had an average life satisfaction score (mean = 23.6 ± 4.09). Illness, childlessness, and loneliness were major factors affecting life satisfaction. Chronic illness and loneliness were the primary reasons for residing in the home. Perceptions of the geriatric home were mixed: 53.8% of participants viewed it negatively due to movement restrictions and lack of privacy, while others appreciated the care provided. Despite some positive perceptions, all participants expressed a desire to leave the home and reunite with family, highlighting the importance of strong family ties for enhancing life satisfaction.
Govender, S.M.; de Jongh, M. 2021. *Identifying hearing impairment and the associated impact on the quality of life among the elderly residing in retirement homes in Pretoria*,* South Africa*	South Africa	Private and state retirement homes	Determine psychological, communication-related, and social impact of hearing impairment on QoL.	Prospective cross-sectional research, quantitative methods.	218 participants; ages 65–98; 79% female	The World Health Organization Quality of Life (WHOQoL) questionnaire was adapted and utilized for the present study.	Hearing difficulties linked to reduced QoL. Hearing aids improve QoL for most users; challenges in social interaction using of hearing aids. Hearing impairment has an impact on the social, psychological and communication aspects thereby reducing QoL.
Oyebade, O.E.; Oyinlola, O.; Asenjo Palma, C. 2024. *Difficult but achievable: medical social workers’ experiences transiting older people from hospital care to nursing home in Nigeria*	Nigeria	Government owned hospital	Explore experiences of medical social workers supporting older people’ transition from hospital to nursing homes.	Descriptive phenomenology, qualitative content analysis.	16 medical social workers with > 2 years’ experience in transition support.	Semi-structured interviews.	Challenges include funding, cultural concerns, family dynamics, and inadequate policies. Calls for community-based nursing care services and better policy support.
Gutiérrez, M.; Tomás, J.M.; Sancho, P.; Galiana, L.; Francisco, E.-H. 2014. *Perception of quality of life in an elderly Angolan sample*	Angola	Nursing homes (lares) and community dwellers.	Analyze differences in perceived health and life satisfaction among elderly Angolans by age, gender, and living situation.	A transversal interview.	1003 elderly persons; majority 60–79 years old, 65.4% female; 76.3% in nursing homes.	Surveys: Satisfaction with Life Scale, Perceived Health Scale.	Higher satisfaction and perceived health among those living with family compared to those in nursing homes. Clear quality of life differences based on age and living situation.
Gerber, A.M.; Botes, R.; Mostert, A.; Vorster, A.; Buskens, E. 2016. *A cohort study of elderly people in Bloemfontein*,* South Africa*,* to determine health-related quality of life and functional abilities*	South Africa	Nursing homes	Impact of chronic diseases on QoL and functional ability.	EQ-6D, ICECAP-O questionnaires.	104 elderly individuals (≥ 65 years)	Using the descriptive system of the SixDimensional EuroQol questionnaire (EQ-6D) and a version of the ICEpop capability measure for older people (ICECAP-O).	Pain and mobility were most affected domains; importance of health promotion, disease prevention, and improving healthcare services for elderly to enhance QoL.
Stuart-Röhm, K.; Clark, I.N.; Baker, F.A. 2024. *Person-centred caregiver singing for people living with dementia in South Africa: A mixed methods evaluation of acceptability*,* feasibility*,* and professional caregivers and experiences.*	South Africa	Care homes.	Acceptability and feasibility of PCCS (Person-centered caregiver singing) in care homes.	Mixed methods data collection.	41 formal caregivers across seven care homes.	A 20-item questionnaire.	PCCS improved residents’ well-being and caregivers’ emotional capacity. Challenges included limited song repertoire and unpredictability of residents.
Brooks, Deborah; Johnston, Sandra; Parker, Christina; Cox, Leonie; Brodie, Melissa; Radbourne, Catherine; MacAndrew, Margaret. 2024. *Elements of Long-Term Care That Promote Quality of Life for Indigenous and First Nations Peoples: A Mixed Methods Systematic Review*	North America (8), South Africa (1), Norway (1), New Zealand (1), and Australia (7)	Nursing and residential care facilities.	QoL and consumer satisfaction for Indigenous and First Nations in LTC.	Mixed methods systematic review.	18 Studies included.	Mixed methods systematic review following the Preferred Reporting Items for Systematic Reviews and Meta-Analyses (PRISMA) reporting guidelines; JBI methods for mixed methods systematic reviews and the ConQual approach to assessing confidence in qualitative evidence synthesized findings.	Culturally safe care, trauma-informed policies, respect for cultural practices, and connection with community were key elements for improving QoL.
Nwakasi, Candidus; Brown, J. Scott; Subedi, Sree; Darlingtina, Esiaka. 2021. *Depression*,* functional disability*,* and accessing health care among older Ghanaians and South Africans: a comparative study based on WHO study on global ageing and adult health (SAGE)*	South Africa	Urban residential care facility.	Social capital and mental well-being.	Quantitative descriptive survey.	103 participants (≥ 60 years)	WHO-5, Kessler-6, OSLO-3, ABS Indigenous Questionnaire, Social Capital Framework.	Moderate mental well-being; participation in activities and primary network predicted well-being.
Ntozini, Anathi; Abdullahi, Ali Arazeem. 2021. *Loneliness and psychological well-being among the elderly in Buffalo City*,* South Africa*.	South Africa	Rented home and home for the aged.	Moderating effect of ethnicity on the relationship between loneliness and psychological well-being.	Hierarchical regression analysis using IBM SPSS.	301 elderly people	Ryff’s Psychological Well-being Scale, UCLA Loneliness Scale.	Ethnicity moderates the impact of loneliness on autonomy, environmental mastery, and self-acceptance. White elderly with low levels of loneliness had higher self-acceptance.
Teka, Alemnesh; Adamek, Margaret. 2014. *‘We Prefer Greeting Rather Than Eating:’ Life in an Elder Care Center in Ethiopia*	Ethiopia	Hospital-like settings (e.g., nursing homes).	Explored the psychosocial needs of older people in a residential elder care center in Ethiopia.	Observation, document review, interviews, focus group discussions.	24 residents, 5 staff	Guiding questions.	Gratitude for shelter despite inadequate provisions; residents yearn for meaningful social interaction. Psychosocial support undervalued.
Hanssen, Ingrid; Kuven, Britt Moene. 2016. *Moments of joy and delight: the meaning of traditional food in dementia care*	South Africa and Norway	Geriatric facilities.	Significance of traditional food in dementia care for institutionalized patients.	Qualitative design, in-depth interviews, hermeneutic analysis.	18 family members, 19 nurses	In-depth interview approach guide.	Traditional food improves cultural identity, joy, belonging, appetite, and quality of life in dementia care. Needs of imperative clinical and moral concern.
De Swart, M.; De Vries, M.; Roos, H.; Joubert, G.; Pienaar, P.E. 2004. *Physical activity knowledge*,* attitudes and practices of the elderly in Bloemfontein old age homes*	South Africa	Old age home.	Determine physical activity knowledge, attitudes, and practices of the elderly.	Descriptive study, pilot questionnaire.	390 residents (81.5% female)	Structured questionnaire.	Good knowledge of physical activity’s influence on quality of life.
Gutiérrez, M.; Tomás, J.M.; Galiana, L.; Sancho, P.; Cebrià, M.A. 2013. *Predicting life satisfaction of the Angolan elderly: a structural model*	Luanda	Old people’s homes.	Predictive power of successful aging components on life satisfaction.	Cross-sectional survey, structural MIMIC model.	1003 elderly (65.4% women)	Satisfaction With Life Scale, Perceived Health Scale, Generativity indicators.	Active engagement with others strongly predicts life satisfaction.
Ramocha, Lesego M.; Louw, Quinette A.; Tshabalala, Muziwakhe D. 2017. *Quality of life and physical activity among older people living in institutions compared to the community*	South Africa	Old age homes.	Measure quality of life and physical activity levels among community-dwelling and institutionalized older people.	Analytic cross-sectional study.	80 participants (40 in each group)	RAND 36, Physical Activity Scale for the Elderly (PASE), Mini Mental State Examination (MMSE).	Community dwellers have higher quality of life and are more physically active than those in old age homes.
Chipps, Jennifer; Jarvis, Mary Ann. 2016. *Social capital and mental well-being of older people residing in a residential care facility in Durban*,* South Africa*	South Africa	Urban residential care facility.	Investigate social capital and mental well-being among older persons.	Quantitative descriptive survey, pilot study.	103 (60 + years, cognitively intact)	WHO-5, Kessler-6, OSLO-3, ABS Indigenous Questionnaire, Social Capital Measures.	Nearly half were mentally unwell; social capital was linked to mental well-being, highlighting the importance of preserving social capital in residential care.
Kago, M.;Kavulya, J.;Mutua, Daniel. 2016. *Influence of institutionalized care on psychosocial well-being of the elderly in Kenya: a case of Nyumba ya wazee Nairobi county*,* Kenya*	Kenya	Public long term care facility	The main objective of this study was to find out the influence of institutionalized care on psychosocial well- being of the elderly in Kenya.	case study research design	118 persons who included the institutions managers, staffs and the elderly persons in Nyumba ya Wazee.	structured questionnaires while interview schedules was administered to the aged people within the Home	Institutionalized care for the old was found to influence psychosocial well-being of elderly. The care given to the elderly was found to be very important because it influences their psychosocial well-being and hence improves the lives of the elderly at large. Institution policy framework was found to influence the psychosocial well-being of elderly. Old person placed in the institution for the aged do have relative caregivers though they are also assigned non-relative caregivers. Further it was clear from this study that old people are comfortable with the policy frame work for the institution and they have good attitude towards their caregivers which influences positively towards their psychosocial well-being.
Oluwagbemiga O. 2016. *Effect of social support systems on the psychosocial well-being of the elderly in old people s homes in Ibadan.*	Nigeria, Ibadan	Residential and non-residential old people’s homes	examine the effect of social supports on the psychosocial well-being of the elderly in selected old peoples’ homes in Ibadan	descriptive survey research design	sample comprised of 122 elderly within the ages of 65 years and above who were purposively drawn from three (3) old people’s homes in Ibadan that operate on residential and non-residential services	Three research instruments were used: Social Support was assessed by the adapted scale of “multidimensional scale of perceived social support” (*r* = 32); psychological well-being was measure by adapted scale of ‘the general psychological well-being index’ (*r* = 82); social well-being was measured by adapted scale of ‘Perceived Social Well-being Questionnaire’(r = 68).	The study found that social support significantly improves the psychosocial well-being of elderly individuals in old age homes. Emotional support, financial support, companionship, and information access were all impactful, with companionship contributing the most, followed by information access, emotional support, and financial support. The findings emphasize the need for a structural framework in old age homes to enhance services, including economic support, positive societal attitudes towards aging, and comprehensive elderly care systems.

### Findings across ecological system levels

Bronfenbrenner’s ecological systems theory was employed as a conceptual and analytical framework to interpret the data rather than as a tool for data collection. The theory provided a structured lens through which to examine how different environmental systems—ranging from immediate interpersonal relationships (microsystem) to broader socio-political structures (macrosystem)—interact to influence the quality of life of older people in long-term care facilities. This theoretical framing guided the organization and analysis of findings by helping to identify the multilevel factors and interactions that shape institutional practices and residents’ everyday experiences (see Table 5 for a summary of findings).

**Table 5:** Summary of key findings across ecological system levels

Ecological Level	Key Findings
Microsystem	Reduced social interaction; importance of caregiver support; facility infrastructure impacts well-being
Mesosystem	Family contact central to QoL; caregiver-family collaboration lacking; caregiver-led activities support engagement
Exosystem	Institutional policies shape psychosocial environment; staff training in gerontology essential; Ubuntu philosophy offers contextual framework
Macrosystem	Cultural misalignment of QoL tools; socio-economic disparities affect access to care; stigma and cultural norms influence well-being

### Microsystems

The microsystem is the innermost layer of Bronfenbrenner’s ecological theory and consists of direct interactions between the individual and their immediate environment. The study conducted by Chipps and Jarvis [53] reveals that older people in residential care experience diminished social capital, which subsequently affects their mental well-being. The separation from significant others and limited interaction with external communities contribute to feelings of social isolation among older people [50, 55–57]. These studies show that relationships could either foster social bonds or exacerbate isolation, depending on the quality of the relationships and participation in group activities within the facility. Those with higher involvement in activities, both inside and outside the facility, have shown better mental well-being [49, 58]. However, low participation in external activities, as noted by Chipps and Jarvis [53], suggests a barrier within the microsystem, which limits opportunities for older people to engage and improve their QoL.

The caregiver’s role within the microsystem is crucial for the older people’ emotional and physical well-being. Stuart-Röhm et al. [59] emphasize the importance of person-centered caregiving, which focuses on recognizing the individual’s uniqueness and meeting their emotional needs. When caregivers engage in meaningful interactions, such as person-centered caregiving through activities such as singing, the mood and cognitive function of the residents improve, thus creating a more supportive microsystem. For example, the paper by Govender and de Jongh [55] highlights how facilitated communication among older people with hearing impairments by caregivers significantly improved the participants’ QoL. In another study conducted in Kenya, older people reported positive impact on their QoL following care given by both care workers and family members who volunteered at the LTC facility [60].

The physical environment of the facility directly influences interactions in the microsystem. Teka and Adamek [54] highlight the lack of basic amenities, such as soap and toilet tissue, in some residential care centers, as negatively affecting the psychosocial well-being of the residents. Other studies highlight repetitive food and inadequate health services as generating dissatisfaction and creating a hostile microsystem where residents feel neglected [51, 54]. Additionally, the restrictive nature of LTC homes similarly made older people feel dissatisfied because they lack the needed privacy and autonomy [51]. Gerber et al. [61] and Kago et al. [60] show that having a private room enhances the older person’s sense of privacy and dignity.

### Mesosystem

The mesosystem in Bronfenbrenner’s ecological model refers to the interactions between different parts of a person’s microsystem. In the context of older residents in LTC facilities, it involves the dynamics between residents, their families, caregivers, and institutional support structures. These interactions play a crucial role in shaping the residents’ overall QoL, mental well-being, and social capital.

Several studies in this review highlight the importance of family relationships to the well-being of older people [51, 52, 54, 57]. Teka and Adamek [54] emphasize that meaningful interaction with family is central to promoting QoL in older residents. As such, many residents express a desire for more frequent contact with family members, which is often restricted in institutional settings [62–64]. As Ntozini and Abdullahi [50] put it, the identified lack of family interaction contributes to feelings of loneliness which negatively affect the psychological well-being of residents. Gutiérrez et al. [56] and Odetola et al. [52] emphasize the decline in life satisfaction among older people in LTC facilities, especially those with poorer health or lacking family ties.

The concept of social capital is crucial in understanding the mesosystem. Chipps and Jarvis [53] discuss how residents in LTC facilities often experience diminished social capital due to separation from their families and community. Social capital, which refers to the resources and support derived from social networks, is significantly linked to residents’ mental well-being [65]. When residents are disconnected from their families or community networks, their sense of support and belonging decreases, leading to decreased well-being [50, 52, 58, 66]. The support structures provided within the LTC facility itself, such as caregiver relationships and organized social activities, attempt to mitigate this loss [54]. This is evidenced by Pienaar et al. [49] who found that caregivers play an essential role in fostering residents’ physical and mental health by encouraging physical activities. The quality of the relationship between residents and caregivers serves as another key component of the mesosystem which directly impacts the residents’ QoL and mental health.

Stuart-Röhm et al. [59] focus on the person-centered care approach, which involves caregivers recognizing residents’ individuality and emotional needs. This approach improves the relationship between residents and caregivers, enhancing mutual well-being and creating a more nurturing environment. Caregivers also influence the residents’ level of engagement in activities that promote social connections. A well-functioning mesosystem requires effective communication between caregivers and the residents’ families. Govender and de Jongh [55] highlight that residents with hearing impairments often face social isolation, but when caregivers actively support their use of hearing aids, social interactions and QoL improve. Similarly, caregivers in Hanssen and Kuven’s [51] study brought traditional foods from home to improve the social and emotional well-being of residents, demonstrating the deep emotional connections that can develop between residents and caregivers in an institution. Kago et al. [60] found that emotional and financial support from families, when combined with high-quality care from the institution, contributed positively to the psychosocial well-being of elderly residents. Families and caregivers must collaborate to provide holistic care, yet this analysis suggests that such cooperation is often lacking. Many families have limited involvement, placing a greater burden on caregivers, which can negatively affect the residents’ psychosocial environment.

### Exosystem

Several studies in the analysis indicate the significant influence of enabling institutional policies on the QoL of elderly residents. Kago et al. [60] highlight the direct impact of institutional policy frameworks on the psychosocial well-being of elderly individuals. For instance, policies that provide emotional support, home repairs, financial support, and social services can significantly improve residents’ lives. This aligns with Bronfenbrenner’s concept of the exosystem, where policies outside the individual’s immediate environment (i.e., management frameworks) shape their lived experience. Ntozini and Abdullahi [50] and Hanssen and Kuven [51] emphasize the need for policies that address cultural diversity within LTC homes. The South African philosophy of *Ubuntu*, which focuses on communal care and interconnectedness, was proposed by Jarvis et al. [56] as a model to combat isolation. Stuart-Röhm et al. [59] emphasize the importance of person-centered care within LTC facilities, particularly for residents with dementia. The study advocates for frameworks that recognize individuality and aim to enhance emotional and social needs. Oluwagbemiga [67] adds that well-structured support systems, including emotional and informational support, and companionship, are essential in improving psychological well-being in LTC. The importance of caregiver training and its influence on the well-being of residents is evident. Kago et al. [60] and Oluwagbemiga [67] stress that caregivers need specialized training in gerontology and mental health to meet the psychological and social needs of the elderly. This reflects Bronfenbrenner’s notion that institutional training programs, though indirectly related to residents, have a profound impact on the quality of care they receive. Odetola et al. [52] concur by recommending the inclusion of geriatric care in nursing and health professional curricula to improve the overall care framework in LTC facilities. Further, Teka and Adamek [54] call for better training of healthcare workers in geriatric care, more flexible policies, and family-friendly environments to enhance life satisfaction of LTC residents.

### Macrosystem

The macrosystem includes broad societal factors, such as cultural norms, economic conditions, and public policies that influence individual and group behaviors. The WHOQOL-OLD instrument, referred to across studies, reflects an international effort to assess QoL among older people [16, 55]. However, the cultural fit of these tools, as suggested by Van Biljon and Roos [26], and in further work conducted by Van Biljon et al. [16], raises concerns. In South Africa, validation in the mother tongue is recommended to better address the cultural relevance for local populations. Cultural differences also emerge regarding food preferences and psychosocial health [54]. Hanssen and Kuven [51] note that traditional food plays a critical role in the well-being of dementia patients in South African LTC facilities. The incompatibility between the food provided and cultural preferences significantly affect emotional and social well-being [51, 54]. The societal perception of aging in sub-Saharan Africa is marked by vulnerabilities, such as poverty, exclusion from health services, and insufficient family support systems [56, 58]. Institutional care appears to carry different meanings across racial and economic divides in South Africa. Ramocha et al. [58] observed that White older persons often enter institutional care with better financial planning, whereas Black older South Africans do so out of destitution. This stark difference suggests that societal views on aging and care are deeply influenced by socio-economic conditions​. Govender and de Jongh [55] further emphasize the stigma associated with conditions such as hearing loss, which can lead to social isolation.

Guided by Bronfenbrenner’s ecological systems theory, the discussion of findings reflects a multilevel understanding of the quality of life of older people in long-term care. The theory enabled us to map how experiences at the individual and interpersonal levels are influenced by institutional policies, community dynamics, and broader structural conditions. This ecological perspective emphasized the interconnectedness of systems and underscored the importance of addressing not only immediate care practices but also systemic factors such as governance, staffing norms, and social policy.

## Discussion

This review mapped and described the existing evidence on the QoL of older people living in LTC facilities in sub-Saharan Africa. The findings highlight key factors influencing QoL, including socio-economic status, access to care, and institutional conditions. By synthesizing the available literature, the review provides a comprehensive overview of the current state of knowledge and identifies critical gaps in research that need to be addressed to improve QoL outcomes for this population. Institutional Ethnography, as developed by Dorothy Smith [68], is a framework that focuses on uncovering the social relations and institutional practices that organize everyday experiences. In the LTC context of older people, this ethnographic approach assists in revealing how institutional structures, policies, and relationships impact the QoL of residents. This scoping review was therefore guided by an IE framework to map out how institutional practices across different ecological systems influence QoL within LTC facilities across sub-Saharan Africa.

One of the key insights from this review is the disjuncture between institutional policies and the day-to-day realities experienced by LTC residents. As documented in multiple studies included in this review, LTC facilities in sub-Saharan Africa often struggle with underfunding, resource shortages, and inadequate caregiver training [30]​. These structural challenges are directly linked to the organization of work within these institutions, where caregivers and staff must navigate multiple demands, often with limited resources. These limited resources notably put a strain on access to essential resources, such as basic amenities (soap and hygiene products) and proper nutrition, and in turn negatively impact the residents’ psychosocial well-being [54]​. This reality illustrates how institutional limitations can shape the everyday lives of residents, leading to feelings of neglect and dissatisfaction.

A further insight is the integral role of caregivers in the well-being of older people. However, inadequately trained staff exacerbate the challenges faced by older people, and can negatively impact the care experience. As Mapira et al. [30] note, caregivers in many LTC facilities across the African region lack specialized training in gerontology and geriatrics, which results in inadequate emotional and physical support for residents. This lack of professional development reflects the broader institutional organization, where staffing needs are not prioritized, and caregivers are left to navigate their roles without the necessary tools for effective care services. In this context of LTC, a typical bed and body approach and culture to care becomes common, where interactions between the staff and residents is often limited to routine work of accomplished tasks, such as only attending to bodily needs of residents and making beds. Such organizational shortcomings have profound consequences for residents living in LTC settings, and directly affect the QoL of residents, including limiting their access to person-centered care practices, disregarding the residents’ psychosocial needs, and diminished humaneness, which can often be infantilizing – all of which are critical considerations for maintaining dignity and emotional well-being [59].

Smith’s IE framework emphasizes the importance of examining how social relations are coordinated within institutions [68]. The findings from this scoping review highlight the role of family, caregivers, and the wider community in influencing residents’ well-being. Social capital, as noted by Chipps and Jarvis [53], is a critical element of QoL in LTC settings, where diminished social interactions and disconnection from family lead to increased feelings of loneliness and isolation​. This suggests that institutional arrangements, such as the noted restrictive visitation policies or inadequate community engagement, hinder the ability of residents to maintain meaningful relationships beyond the bounds of the residential institution. The implication here is that older people’s previously established familial and social relations are weakened, and even broken down. The institutional regulation of family visits and participation in external community activities exemplifies how organizational structures limit social capital and negatively impact mental well-being.

External social capital is of great importance for older people, as these external relations sustain feelings of autonomy among older people in the context of restrictive LTC institutions. The same social capital could address issues identified regarding culturally appropriate food, and, more broadly, sense of support and belonging. Visitors often bring preferred food to the facility for the residents, and relational networks could assist in addressing loneliness and a diminished sense of support and belonging – all of which impact the residents’ both physically and emotionally. It can thus be argued that institutional limitations create conditions for LTC residents which negatively impact them, generating increased dissatisfaction and poorer QoL. Residents are often without power to change such institutional practices. Therefore, these findings are expected to inform those working in LTC facilities to create practices that preserve external bonds with family members, friends and volunteers.

The review underscores the importance of fostering caregiver-resident relationships. Studies by Govender and de Jongh [55] show that when caregivers actively support residents, particularly those with impairments such as hearing loss, there is a notable improvement in residents’ QoL​. This aligns with Smith’s argument that the organization of care work is deeply interconnected with the institutional processes that structure LTC [72]. Caregivers’ ability to engage with residents on a personal level – whether through assisting with communication aids or encouraging participation in social activities – demonstrates the potential for improving QoL when care is organized in a way that prioritizes the relational needs of residents.

Furthermore, institutional policies regarding staffing ratios, funding allocations, and caregiving responsibilities significantly shape the day-to-day experiences of both caregivers and residents. Studies included in the review advocate for a reform in policy with an aim to address underfunding of LTC facilities and improve the working conditions of caregivers [60]. Such reforms are necessary to ensure that institutional structures support, rather than hinder, the ability of caregivers to deliver high-quality, personalized care. IE thus provides a lens through which we can critically analyze how policies are enacted within LTC facilities, and how these policies shape both the organizational practices and the QoL of older people in LTC facilities.

### Limitation

While these findings largely reflect challenges identified in the South African context, similar structural and institutional barriers may be present elsewhere in sub-Saharan Africa.

However, the generalizability of the findings is limited by the geographic concentration of studies with two-thirds of the included research was conducted in South Africa. This imbalance may reflect disparities in research infrastructure and publication output across the region, yet it also limits insights into diverse care contexts in countries with different socio-political and healthcare systems. Moreover, the restriction to English-language publications may have introduced language bias, potentially excluding relevant research published in French, Portuguese, or Arabic, which are widely spoken in various sub-Saharan African countries. These constraints should be considered when interpreting the findings and underscore the need for further research across a broader range of linguistic and geographic settings.

## Conclusions

This scoping review provides a foundational understanding of the current knowledge landscape on QoL among older people in LTC facilities in sub-Saharan Africa. The review reveals that while QoL is widely recognized as a core outcome of LTC, it is often assessed through resident satisfaction, with measurement tools such as WHOQoL and ICECAP-O commonly employed. However, the review also highlights a limited and uneven evidence base, with the majority of studies concentrated in South Africa and a reliance on qualitative methodologies. Findings indicate that QoL is shaped by a complex interplay of factors at multiple ecological levels. These include the physical and emotional quality of care, relationships with staff and family, institutional culture, and broader policy and socio-economic structures. Rigid institutional routines and the absence of enabling policy environments restrict residents’ autonomy and well-being, reinforce conformity through daily interactions with care staff. These insights underscore the need for a comprehensive, contextually appropriate LTC policy, one that promotes standardized, person-centered care while addressing workforce shortages, inequities in resource allocation, and the fragmentation of services. Recognizing the pivotal role of family and community networks in enhancing QoL, such policy should also strengthen intersectoral partnerships and expand support for home- and community-based care. Although the review draws primarily from South African literature, similar structural and relational barriers are likely present across other Sub-Saharan African countries. To develop inclusive and responsive LTC systems across the region, there is a pressing need for expanded, multilingual research that captures diverse socio-cultural, linguistic, and care contexts.

## Supplementary Information

Supplementary Material 1.

## Data Availability

No datasets were generated or analysed during the current study.

## Abbreviations

ADL: Activities of Daily Living
QOL: Quality of Life
IE: Institutional Ethnography
IPA: Interpretative Phenomenological Analysis
LTC: Long-Term Care
PCC: Population, Concept, Context
PRISMA-P: Preferred Reporting Items for Systematic Review and Meta-Analysis Protocols
PRISMA-ScR: PRISMA extension for a Scoping Review
WHO: World Health Organization
WHOQoL: World Health Organization Quality of Life
